# Evaluation of Different Dentifrice Compositions for Increasing the Hardness of Demineralized Enamel: An in Vitro Study

**DOI:** 10.3390/dj7010014

**Published:** 2019-02-04

**Authors:** Pedro Henrique Cabral Oliveira, Marcia Regina Cabral Oliveira, Luiz Henrique Cabral Oliveira, Ravana Angelini Sfalcin, Marcelo Mendes Pinto, Ellen Perin Rosa, Alessandro Melo Deana, Anna Carolina Ratto Tempestini Horliana, Paulo Francisco César, Sandra Kalil Bussadori

**Affiliations:** 1 Department of Biophotonics Applied to Health Sciences, University Nove de Julho (UNINOVE), Rua Vergueiro 235/249, Liberdade, São Paulo 01504-001, Brazil; cabral-oliveira@live.com (P.H.C.O.); marcia-cabral@uni9.pro.br (M.R.C.O.); luizhenrique.c.o@icloud.com (L.H.C.O.); ravanasfalcin@uni9.pro.br (R.A.S.); mmpdent@uni9.pro.br (M.M.P.); ellen_perim@hotmail.com (E.P.R.); amdeana@uni9.pro.br (A.M.D.); acrth@uni9.pro.br (A.C.R.T.H.); 2 Department of Biomaterials and Oral Biology, University of São Paulo, Av. Professor Lineu Prestes, 2227 (Cidade Universitária), São Paulo 05508-900, Brazil; paulofc@usp.br

**Keywords:** arginine, fluoride, dental enamel, dental caries, demineralization, tooth remineralization

## Abstract

This study aimed to evaluate microhardness of a dentifrice containing fluoride and arginine compared to a positive control (fluoride only) and a negative control (no fluoride) on sound and demineralized bovine enamel surfaces. Specimens were randomly assigned to different treatments that included daily pH cycling and brushing three times a day with one of the following dentifrices (*n* = 8): Neutraçucar (arginine and fluoride), Colgate Total 12 (fluoride) and My First Colgate (no fluoride). Enamel carious lesions were artificially created one week before the beginning of these treatments (demineralized bovine enamel (DE) groups). The same groups were also tested in sound enamel (sound bovine enamel (SE) groups). Microhardness was measured at baseline and after one, two, and five weeks of treatment using a Knoop indenter. Statistical analysis involved two-way Analysis of Variance (ANOVA) and Tukey’s test. After five weeks, both Total 12 and Neutraçucar had increased the microhardness of DE specimens (*p* < 0.05). Only Neutraçucar had increased the microhardness of the sound enamel after five weeks of treatment. Thus, it could be concluded that arginine-based dentifrices increase the microhardness of sound and demineralized bovine enamel surfaces.

## 1. Introduction

Despite the daily use of fluoride dentifrices by a large portion of the population around the world, dental caries continues to be the most prevalent oral condition and untreated carious lesions remain a serious public health problem [1]. Caries is a multifactorial and dynamic disease [2,3] characterized clinically by mineral loss of dental hard tissues. Bacteria forming dental plaque can metabolize carbohydrates to produce lactate and other acids that demineralize the enamel, a process in which calcium and phosphate are dissolved away from hydroxyapatite crystals within the enamel. However, the development of carious lesions can be stopped or reversed when the acid challenge is removed and saturated saliva returns calcium, phosphate, and fluoride minerals to the enamel surface, thereby repairing the hydroxyapatite structure [4]. The standard treatment of caries consists of re-balancing the demineralization and remineralization processes and involves control of risk factors such as frequent ingestion of dietary carbohydrates, control of dental plaque formation by tooth brushing, and exposure of dental hard tissues to fluoride by using dentifrices and other oral products containing fluoride. Dentifrices containing 1.5% arginine, 1450 ppm fluoride and sodium monofluorophosphate in insoluble calcium have been recently introduced to the market. Studies performed with these dentifrices containing arginine suggest that arginine can positively affect plaque metabolism and decrease caries risk [1,5,6,7]. Arginine is a semi-essential amino acid found in different foods and beverages such as milk, milk by-products, beef, poultry, shellfish, cereals, and dried fruits, and it is considered safe for use in dentifrices. Insoluble calcium combined with 1.5% arginine may be presented in the form of calcium carbonate or dehydrated calcium dehydrated. Certain oral microorganisms such as *Streptococcus sanguinis* metabolize arginine to produce ammonia, which neutralizes plaque acids and favors plaque pH homeostasis [8]. Specifically, ammonia production via arginine metabolism prompts a neutral environmental pH that is less favorable to the outgrowth of acid-producing cariogenic bacteria, thus reducing caries risk. In fact, clinical studies have revealed a positive correlation between arginine metabolism in oral biofilms and an absence of caries activity in adults and children [9,10,11]. The literature reports greater effectiveness regarding the prevention and arrestment of carious lesions when arginine is combined with fluoride in comparison to the use of dentifrices containing fluoride alone [12,13,14]. However, little information is available on the effect of the combination of arginine and fluoride in the demineralization and remineralization processes that will ultimately affect the hardness of sound and demineralized enamel. In this way, the aim of the present in vitro study was to evaluate the effect of treatment with different dentifrices on surface hardness of sound and demineralized bovine enamel. A dentifrice containing 1.5% arginine, 1450 ppm of fluoride, and sodium monofluorophosphate in insoluble calcium was compared to a conventional dentifrice containing 1450 ppm of fluoride and a dentifrice without fluoride. The null hypothesis was that the dentifrice containing fluoride and arginine compared to a positive control (fluoride only) and a negative control (no fluoride) on sound and demineralized bovine enamel surfaces would not decrease the Knoop microhardness of bovine dental enamel after five weeks of treatment.

## 2. Materials and Methods

*Experimental design*—The surface Knoop microhardness of specimens of sound bovine enamel (SE) and demineralized bovine enamel (DE) was measured at baseline and after one, two, and five weeks of treatment. Treatment involved daily pH cycling and brushing three times a day with one of the following dentifrices: Neutraçucar (1.5% arginine and 1450 ppm fluoride—Colgate, Colgate-Palmolive Company, Sao Paulo, SP, Brazil), Colgate Total 12 (1450 ppm fluoride—Colgate, Colgate-Palmolive Company, Sao Paulo, SP, Brazil) and My First Colgate (no fluoride—Colgate, Colgate-Palmolive Company, Sao Paulo, SP, Brazil). The complete composition of each dentifrice is described in Table 1. The six study (*n* = 8) groups are listed in Table 1. 

*Specimen preparation*—Our country defines animal experimentation as procedures conducted with live animals. Since all tooth samples were obtained post mortem from discards of animals raised for commercial slaughter, this study did not require the approval of an animal research ethics committee. Forty-eight recently extracted bovine teeth were kept in 0.1% thymol at 4 °C. The buccal surfaces of all teeth were sequentially abraded with silica carbide water sandpaper with grits of 400, 600, and 1200 (Buehler SA, Joinville, SC, Brazil), followed by polishing with a felt disc and diamond paste with grits of 3, 2, 1, and 0.5 μm (FOX Foco Tools, SP, Brazil) for preparation for the microhardness analysis. Enamel blocks measuring 7 × 4 × 4 mm were cut from the polished buccal surfaces using double-sided diamond discs (7020, KG Sorensen, SP, Brazil) at low speed and under cooling (Kavo, SC, Brazil). The enamel blocks were covered with two layers of acid-resistant nail varnish (COLORAMA, L’Oréal, SP, Brazil), leaving a window of sound enamel surface exposed as previously described [14]. The specimens were randomly distributed among the six groups (*n* = 8) (Table 1). Specimens were kept in a stove at 37 °C. To produce the specimens of demineralized enamel, blocks were submitted to pH cycling for 30 days. This demineralizing pH cycling process consisted of daily immersion in 5 ml of a demineralizing solution (H_2_O, KOH, CH_3_COOH, C_2_H_3_NaO_2_, H_3_PO_4_; pH 3.5–4.0; Fórmula&Ação, São Paulo, Brazil) for 18 h followed by immersion in 5 ml of a remineralizing solution (H_2_O, HCL, KOH, CaCl_2_, (HOCH_2_)_3_CNH_2_; pH 7; Fórmula&Ação, São Paulo, Brazil) for 6 h [15,16]. The specimens of both sound enamel (Neutra SE, Total SE and My First SE) and demineralized enamel (Neutra DE, Total DE and My First DE) were also submitted to daily pH cycling throughout the study period to simulate the cariogenic challenge that occurs clinically in a patient with a high risk of caries [15,16]. For this daily pH cycling, the specimens were immersed in 5 ml of the aforementioned demineralizing solution (DES) for 6 h, followed by abundant rinsing with distilled water and then immersion in 5 ml of the aforementioned remineralizing solution (RE) for 18 h at 37 °C on a shaking table (MacLab, Jacareí, SP, Brazil).

*Treatment protocol*—To simulate tooth brushing, the surfaces of all specimens were brushed three times a day for five weeks with a regular soft toothbrush (Care 5000, Oral B, P&G, Brazil) for 15 s at approximately 40 rotations per minute (rpm) and with use of the tested dentifrices in an aqueous solution (3:1). Table 2 shows the dentifrices used and their composition.

*Surface microhardness analysis*—Surface microhardness was measured using a Knoop indenter (50 g for 20 s) and a microhardness test machine (HMV, Shimadzu, Japan). The specimens were divided into quadrants, with one quadrant used at each evaluation time. The indentations were performed beginning with the central quadrant and avoiding areas near. The fifth reading was performed at a distance of 500 μm from the first indentations in Quadrant 1 to ensure that the same area was not measured more than once. Five indentations were made which were separated by a distance of 100 μm, and the mean of the indentations was considered in the analysis [16]. Surface microhardness was measured on five occasions: immediately after the preparation of the samples (baseline) and immediately after the induction of enamel lesions for Neutra DE, Total DE and My First DE, as well as after one, two, and five weeks of treatment.

*Statistical analysis*—The data were compiled in an Excel spreadsheet and generalized estimation equations were performed with normal marginal distribution and an identity link function, supposing a correlation matrix among evaluation times. Two-way Analysis of Variance (ANOVA) (factors: treatment and time) and Tukey’s test were used for the statistical analysis.

## 3. Results

**Figure 1:**
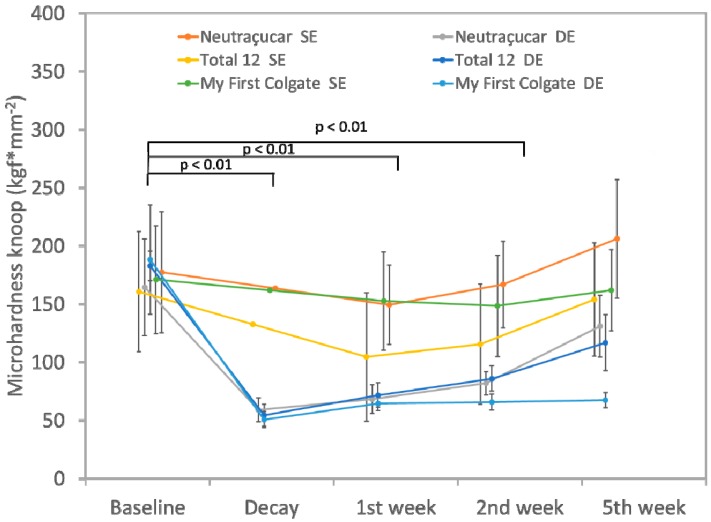
Means of Knoop microhardness of all groups. Error bars represent standard deviation.

Figure 1 shows that there was a gradual increase in the microhardness of DE and SE specimens after brushing with all three dentifrices tested. The two-Way ANOVA shows statistically significant differences in both treatments (*p* = 0.0003) and periods (*p* = 0.0015). Regarding the DE groups (tested in the demineralized enamel), the baseline statistically differs from the decay because there was a demineralization process (*p* < 0.01, Tukey’s test). Electron micrograph of experiment can be found in the supplementary. The fluoride-only dentifrice significantly increased the microhardness of demineralized specimens after one week of treatment. However, in the second week of treatment, Neutraçucar produced a significant increase in surface microhardness while Total 12 gave results similar to those of the first week of treatment (*p* < 0.01, Tukey’s test). Regarding the sound enamel, all groups showed a suitable microhardness and after treatment with the dentifrices, all groups showed an increase in microhardness after five weeks. Moreover, the Neutraçucar SE group differs statistically from the following groups: Neutraçucar DE (*p* < 0.01, Tukey’s); Total 12 DE (*p* < 0.01, Tukey’s) and My First Colgate DE (*p* < 0.01, Tukey’s). The My First Colgate SE group differs statistically from Neutraçucar DE (*p* < 0.05, Tukey’s); Total 12 DE (*p* < 0.05, Tukey’s); and My First Colgate DE (*p* < 0.01, Tukey’s). All other treatments presented statistically similar results.

## 4. Discussion

The anti-caries benefits of dentifrices containing fluoride is well documented in the literature and widely accepted by the public [17,18,19]. More recently, a new technology involving a combination of 1.5% arginine, insoluble calcium, and 1450 ppm of fluoride has demonstrated greater anti-caries efficacy in comparison to dentifrices with 1450 ppm of fluoride alone [1,7]. In the present study, the remineralization capacity of dentifrices containing fluoride, fluoride and arginine, and no fluoride/arginine on sound and demineralized bovine enamel was tested. After five weeks of treatment, mean microhardness values of sound and demineralized enamel indicated gains in tooth minerals, especially for those specimens treated with the fluoride and arginine dentifrice and the fluoride-only dentifrice. The fluoride-only dentifrice significantly increased the microhardness of demineralized specimens after one week of treatment. However, in the second week of treatment, Neutraçucar produced a significant increase in surface microhardness while Total 12 gave results similar to those of the first week of treatment. Thus, the null hypothesis can be rejected as the dentifrice containing arginine and fluoride was able to increase the Knoop microhardness of bovine enamel after five weeks of treatment. We did not include a negative control consisting of just pH cycling without dentifrice treatment, since we did not use any product that interferes with the DES and RE processes. Hence, we consider “My First DE” and “My First SE” as control groups [14]. In corroboration with our findings [20], it was demonstrated that new toothpaste containing arginine provides greater anti-caries benefits than a conventional toothpaste. Also, it has been demonstrated as arresting and reversing the effects of active coronal caries lesions in children compared with positive controls [13].

We also demonstrated a promising anti-caries effect on sound enamel (SE groups), with an increase in microhardness after two weeks of use of the dentifrice containing arginine (173.24 KNH) and the dentifrice containing fluoride only (87.96 KNH). It is important to evaluate SE microhardness because it indicates preventive loss of minerals. Moreover, the acid challenge with pH cycling also demonstrated higher microhardness of DE and SE treated with 1.5% arginine (Neutraçucar, Colgate^®^) as well as the dentifrice containing fluoride only (Colgate Total 12, Colgate^®^) after five weeks of treatment (Figure 1).

Free calcium ions in the oral environment make the saliva super-saturated and favor remineralization, despite the negative charge in demineralized hydroxypatite crystals. The reserve of fluoride in the saliva, acquired film, and hard tissues is another important factor for the occurrence of remineralization. The oral retention of fluoride in a topical regimen is associated with the efficacy of anti-caries prevention [21,22]. There is some evidence indicating that dentifrices containing arginine do not decrease the adhesion of adhesive materials [23]. Moreover, most mineralized enamel favors adhesion and residua in the presence of an arginine interface and may be an additional benefit of preventing the interface and minimizing future exchange restoration. Further studies should investigate this additional effect.

One limitation of this study is that we have tested bovine enamel instead of human teeth. Physical properties and chemical and morphological composition differences between these two types of teeth must be considered when interpreting the results of this study [24], although some studies have proven that bovine enamel is quite similar to that of humans [25,26].

Within the limitations of the present study, Neutraçucar and Total 12 showed higher values of microhardness after five weeks of treatment. Moreover, further experimental and clinical studies regarding remineralization of early enamel caries lesions and prevention methods using Neutraçucar dentifrices are required.

## 5. Conclusions

Within the limitations of this study, it can be concluded that both Total 12 and Neutraçucar increase microhardness of demineralized specimens after five weeks of treatment and that only Neutraçucar increases the microhardness of sound enamel. Thus, Neutraçucar (an arginine based-dentifrice) should be designated for daily toothbrushing as an alternative preventive method in addition to fluoride-only dentifrices.

## Figures and Tables

**Table 1:** Study groups by type of enamel specimen and dentifrice tested.

Study Groups	Type of Enamel	Main Components of the Dentifrice	Commercial Name
Neutra DE	DE	1.5% arginine, insoluble calcium compound, 1450 ppm fluoride as sodium monofluorophosphate.	Neutraçucar Colgate^®^
Neutra SE	SE	1.5% arginine, insoluble calcium compound, 1450 ppm fluoride as sodium monofluorophosphate.	Neutraçucar Colgate^®^
Total DE	DE	1450 ppm fluoride	Colgate Total 12^®^
Total SE	SE	1450 ppm fluoride	Colgate Total 12^®^
My First DE	DE	No Fluoride	My First Colgate^®^
My First SE	SE	No Fluoride	My First Colgate^®^

**Table 2:** Composition and batch number of dentifrices tested in this study. Also shown are the composition of the demineralizing and remineralizing solutions used in this study.

Name	Composition	Batch Number
Demineralizing solution	Acetic acid, Ca(OH)_2_, sodium acetate, phosphoric acid, KOH or HCl in order to regulate pH, and distilled water up to complete 1 l	NR 3046-1
Remineralizing solution	Hydrochloric acid, Ca(OH)_2,_ calcium chloride, tris buffer, KOH or HCl in order to regulate pH, and distilled water up to complete 1 l	NR 3046-0
Total 12	H_2_O, glycerin, sorbitol, hydrated silica, sodium lauryl sulphate, copolymer PVM/MA, cellulose gum, sodium hydroxide, propylene glycol, carrageenan, triclosan, sodium saccharin, sodium fluoride 0.32% (1450 ppm)	5280BR123B
Neutraçucar	Calcium cabonate H_2_O, glycerin, arginine (1.5%), sodium bicarbonate, sodium lauryl ether sulphate, monofluoride phosphate (1.1%, 450 ppm), aroma, cellulose gum, sodium pyrophosphate, benzylic ethanol, saccharin, sodium hydroxide	L3331001021
My first Colgate	H_2_O, sodic sarcina, sorbitol, poloxamer 407, cellulose gum, aroma, citric acid	5026USC11A

## Supplementary Materials

The following are available online at https://www.mdpi.com/2304-6767/7/1/14/s1, Figure S1. Electron micrograph of normal bovine dental enamel magnified 300× and 10000×. Figure S2. The microhardness calibration can be visualized as an indentation of ~1N. This microscopic image was captured in a microhardness program (40×). Figure S3. The microhardness indentation of normal bovine dental enamel. These microscopic images were captured in a microhardness program (A) 40×, (B) 20×. Figure S4. The microhardness indentation of caries lesion bovine dental enamel. These microscopic images were captured in a microhardness program (A) 40×, (B) 20×. Figure S5. The microhardness indentation of caries lesion bovine dental enamel treated with Colgate 12. This microscopic image was captured in a microhardness program (20×). Figure S6. The microhardness indentation of caries lesion bovine dental enamel treated with non-fluoride dentifrice This microscopic image was captured in a microhardness program (40×). Figure S7. The microhardness indentation of caries lesion bovine dental enamel treated with Neutraçucar This microscopic image was captured in a microhardness program (40×).
