# Supplementary material for: Evaluation of Different Dentifrice Compositions for Increasing the Hardness of Demineralized Enamel: An in Vitro Study
Source: Dent J (Basel). 2019 Feb 4;7(1):14. doi: 10.3390/dj7010014 (PMC6473709; doi:10.3390/dj7010014)
Supplement: Supplementary file 1 [file dentistry-07-00014-s001.pdf]

Supplementary Materials: Evaluation of Different Dentifrice Compositions for Increasing the Hardness of Demineralized Enamel: An in Vitro Study

Pedro Henrique Cabral Oliveira [1], Marcia Regina Cabral Oliveira [1], Luiz Henrique Cabral Oliveira [1], Ravana Angelini Sfalcin [1], Marcelo Mendes Pinto [1], Ellen Perin Rosa [1], Alessandro Melo Deana [1], Anna Carolina Ratto Tempestini Horliana [1], Paulo Francisco César [2] and Sandra Kalil Bussadori [1,*]

Surface analysis

Figure 1 displays the same region of normal bovine dental enamel magnified 300× and 10000×. The topography of the enamel is uniform, with the absence of eroded areas as well as the presence of tiny cracks, due to the method employed. The larger magnification illustrates the natural surface arrangement.

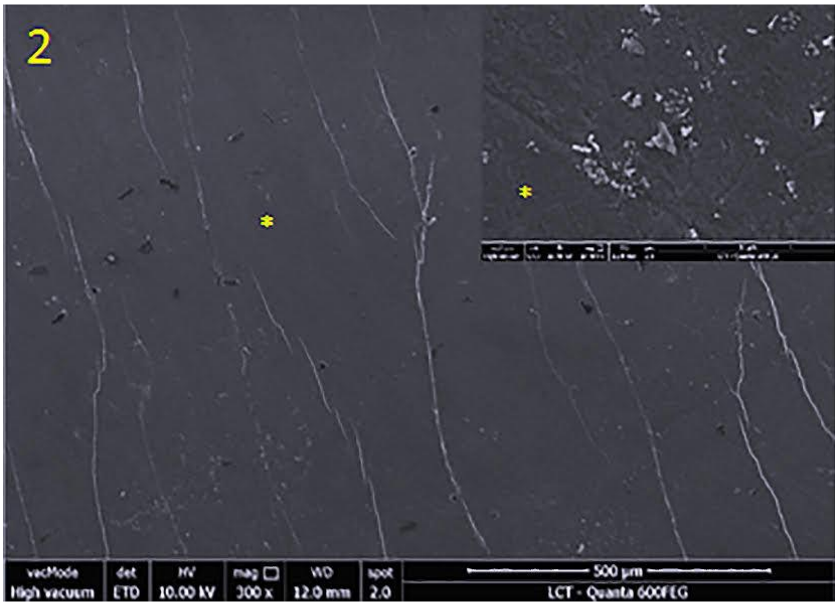

Figure S1. micrograph of normal bovine dental enamel magnified 300× and 10000×. * exact local of magnification.

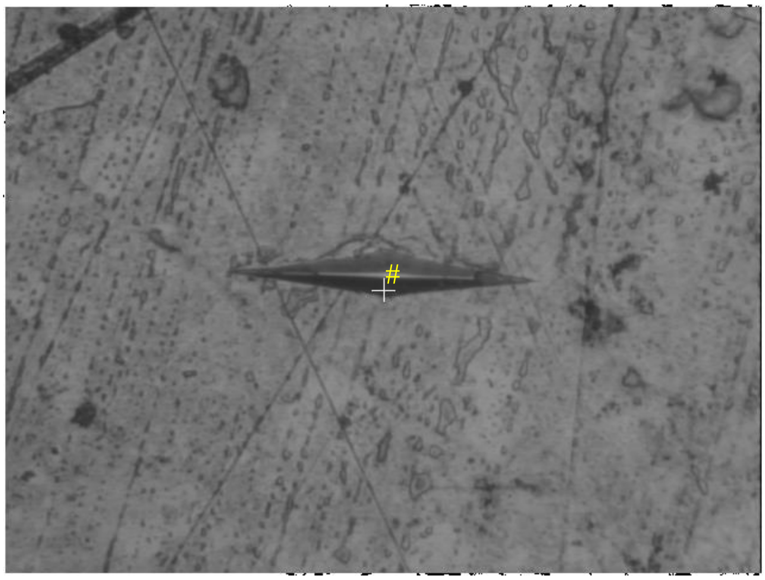

Figure S2. The microhardness calibration can be visualized as an indentation of ~1N. This microscopic image was captured in a microhardness program (40×). # indentation

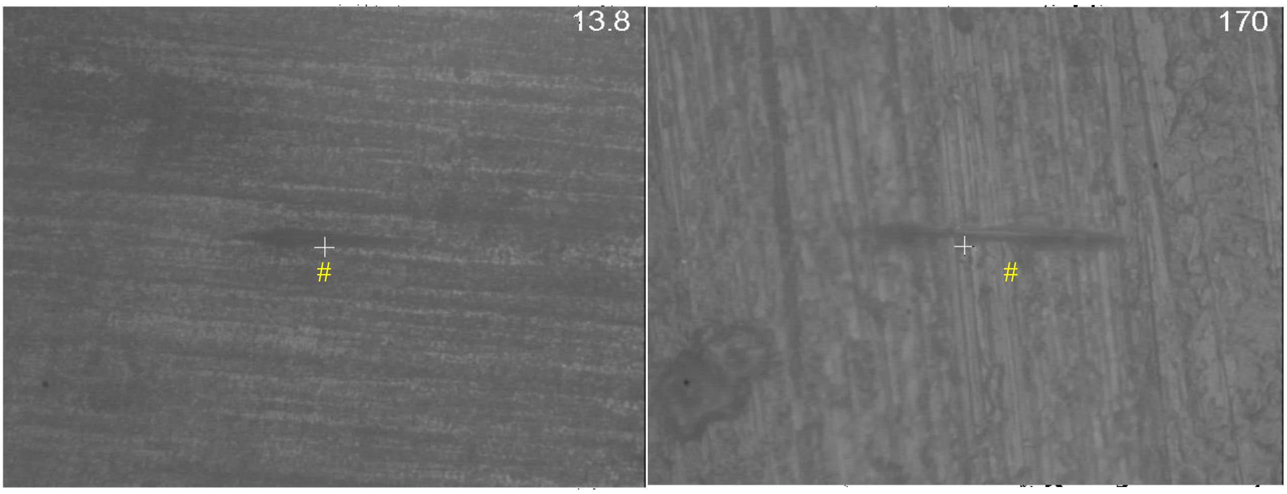

A B

Figure S3. The microhardness indentation of normal bovine dental enamel. These microscopic images were captured in a microhardness program (**A**) 40×, (**B**) 20×. # indentation

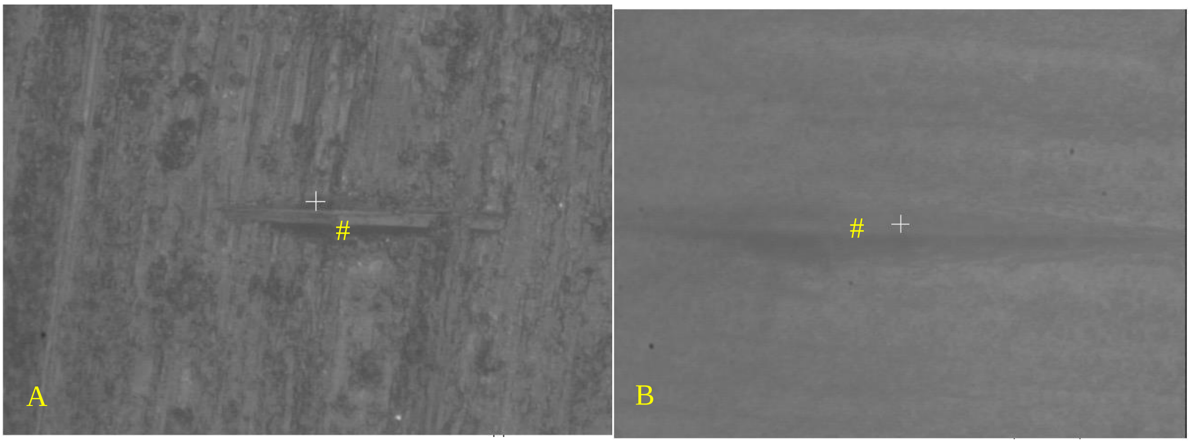

Figure S4. The microhardness indentation of caries lesion bovine dental enamel. These microscopic images were captured in a microhardness program (A) 40×, (B) 20×. # indentation

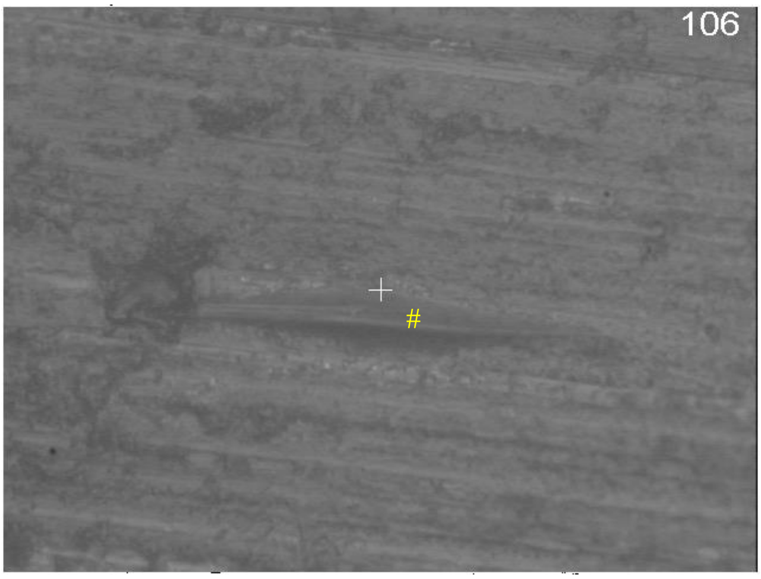

Figure S5. The microhardness indentation of caries lesion bovine dental enamel treated with Colgate 12. This microscopic image was captured in a microhardness program (20×). # indentation

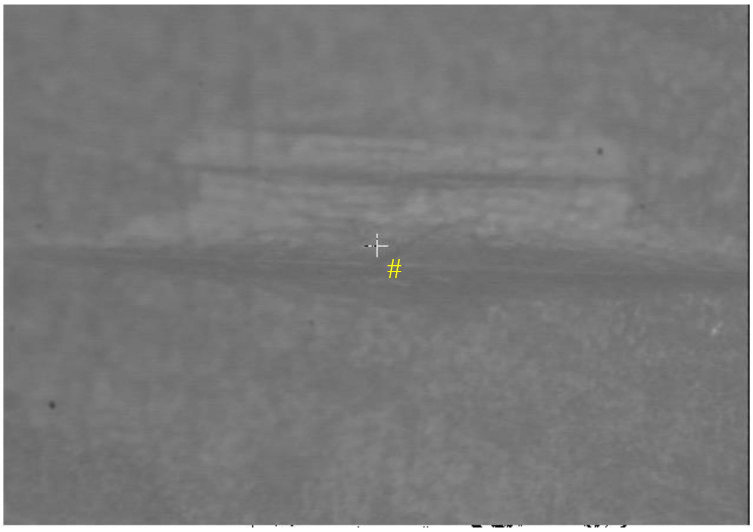

Figure S6. The microhardness indentation of caries lesion bovine dental enamel treated with non-fluoride dentifrice This microscopic image was captured in a microhardness program (40×). # indentation

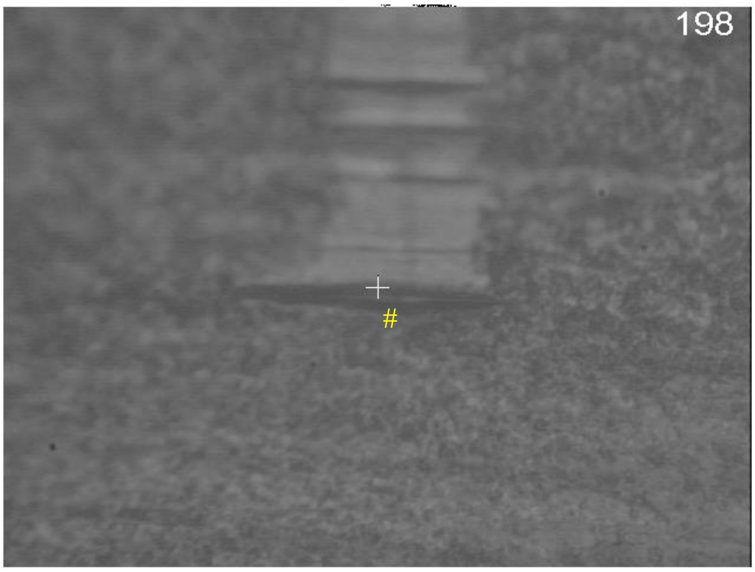

Figure S7. The microhardness indentation of caries lesion bovine dental enamel treated with Neutraçucar This microscopic image was captured in a microhardness program (40×). # indentation
